# The Therapeutic Potential of Antimicrobial Peptides Isolated from the Skin Secretions of Anurans of the Genus *Boana* in the Face of the Global Antimicrobial Resistance Crisis

**DOI:** 10.3390/toxins17070312

**Published:** 2025-06-20

**Authors:** Priscila Mendes Ferreira, Fabiano Fagundes Moser da Silva, Joyce Silva dos Santos, Brunna de Oliveira Silva, Carlos José Correia de Santana, Osmindo Rodrigues Pires Júnior, Wagner Fontes, Mariana S. Castro

**Affiliations:** 1 Laboratory of Toxinology, Physiological Sciences Department, Biological Sciences Institute, University of Brasilia, Brasilia 70910-900, DF, Brazil; primendesf@gmail.com (P.M.F.); fabiano.biomedico@gmail.com (F.F.M.d.S.); joycee.silva1709@gmail.com (J.S.d.S.); brunna.oliveira014@gmail.com (B.d.O.S.); osmindo@unb.br (O.R.P.J.); 2 Laboratory of Protein Chemistry and Biochemistry, Cellular Biology Department, Biological Sciences Institute, University of Brasilia, Brasilia 70910-900, DF, Brazil; wagnerf@unb.br

**Keywords:** Anurans, *Boana*, skin secretion, antimicrobial resistance crisis, antimicrobial peptides, therapeutic properties

## Abstract

Microorganisms play a dual role in human health, serving as both essential allies and serious threats. Their association with infections led to the development of antimicrobials like penicillin, which revolutionized medicine. However, the emergence of antimicrobial resistance (AMR) has created a global health crisis, rendering many treatments ineffective due to pathogen mutations and acquired resistance mechanisms, particularly among ESKAPE pathogens. This resistance increases morbidity, mortality, and healthcare costs, exacerbated by antibiotic overuse and globalization. Biofilms and sepsis further complicate treatment. Addressing AMR requires new therapies, rational antibiotic use, and innovative approaches for drug discovery. Coordinated global action is essential to ensure future access to effective treatments. Antimicrobial peptides (AMPs) derived from *Boana* species (Anura, Hylidae) represent a promising alternative in the fight against AMR. These peptides exhibit activity against multidrug-resistant pathogens. Unlike conventional antibiotics, *Boana* peptides act through a broad mechanism that limits resistance development. Their ability to disrupt bacterial membranes and modulate immune responses makes them ideal candidates for the development of new treatments. These peptides may offer valuable alternatives for treating resistant infections and addressing the global AMR crisis.

## 1. Introduction

Microorganisms play a dual role in human health—as both essential allies and serious threats. Since the pioneering work of Leeuwenhoek, whose innovations laid the foundation for microbiology, our understanding of their role in human disease has significantly expanded, particularly in identifying them as causative agents of infections [1]. To combat microbial infections, antimicrobial agents have become a cornerstone of treatment strategies globally. The discovery of the first antibiotic, penicillin, drastically reduced infectious diseases and saved millions of lives, especially during World War II. Following this discovery, Sir Alexander Fleming warned of the potential for the emergence of bacterial resistance to antibacterial treatments, and soon cases of treatment failures were reported [2].

Multidrug-resistant organisms now spread rapidly across regions and continents through humans, animals, and water [3]. In the 21st century, with globalization and urbanization, treatments can no longer confine resistant strains solely to healthcare settings. Many infectious diseases have become increasingly difficult to control. Major surgeries pose a greater risk due to infection complications. Resistant strains now circulate not only in clinical settings but also in the environment—via agriculture, livestock, and the general population [4].

## 2. Antimicrobial Resistance

Antimicrobial resistance (AMR) refers to the ability of microorganisms—including bacteria, fungi, and parasites—to withstand the effects of drugs designed to eliminate them, such as antibiotics, antifungals, and antiparasitics. This occurs through mutations or the acquisition of resistance genes, allowing pathogens to survive treatments that were once effective [5]. AMR is one of the greatest threats to global health, as it compromises the ability to treat infections, leading to increased morbidity, mortality, and healthcare costs [6].

This growing resistance has intensified the need for new antimicrobial agents. The World Health Organization (WHO) has highlighted this crisis by listing priority pathogens urgently requiring new antibiotics [7]. The ESKAPE pathogens—*Enterococcus faecium*, *Staphylococcus aureus*, *Klebsiella pneumoniae*, *Acinetobacter baumannii*, *Pseudomonas aeruginosa*, and *Enterobacter* spp.—are particularly concerning. In response, researchers are exploring alternative strategies, including adjuvant therapies to boost antibiotic efficacy and the discovery of novel natural and synthetic antimicrobial compounds [7].

The increasing threat of AMR in pathogens like *Pseudomonas aeruginosa*, for instance, presents a major challenge to global public health. *P. aeruginosa* is a Gram-negative bacterium capable of resisting a broad range of antibiotics. This resistance is driven by mechanisms such as efflux pump expression, target site modification, and the production of degrading enzymes like β-lactamases. These bacteria also form biofilms, which hinder antibiotic penetration and promote persistent infection. Given the complexity of resistance in *P. aeruginosa*, new strategies such as antimicrobial peptides (AMPs), bacteriophage therapy, and quorum sensing inhibition—which disrupts bacterial communication required for biofilm formation—are being investigated [8]. AMR not only complicates infection management but also increases treatment duration, healthcare costs, and patient morbidity and mortality. This impact is especially critical in hospital settings, where resistant infections lead to longer hospitalizations and the need for more aggressive interventions. Overuse and misuse of antibiotics are key drivers of resistance. Addressing this requires policies promoting rational use, public education to discourage self-medication, and stronger surveillance and infection control in healthcare settings [8,9].

Millions of deaths from infections occur each year, and treatment failure cannot always be attributed to genetic resistance alone. Fundamental mechanisms, such as biofilm formation and sepsis, play crucial roles in clinical scenarios where antibiotics are ineffective. Biofilms, which are implicated in approximately 65% of infections, confer adaptive resistance by limiting drug penetration and enhancing microbial persistence. Sepsis accounts for nearly 20% of global mortality. Despite being the first-line treatment, antibiotics fail in approximately 23% of cases. Emerging research also highlights the role of host microbiota and immune-modulating therapies as adjunct strategies to address treatment failure. The limited availability of effective therapies and the potential inefficacy of new antibiotics underscore the urgent need to develop new anti-infective agents that directly address therapeutic failure, to mitigate this persistent and increasingly severe problem [10].

Ultimately, developing new therapeutic strategies to combat AMR is critical. In addition to discovering novel antimicrobial compounds, combining therapies with different mechanisms of action can help minimize resistance emergence. Another promising approach involves modifying existing molecules to overcome resistance. Sustained research and innovative strategies are vital to counter this escalating threat and safeguard global public health [7].

The development of new antibiotics is a lengthy and costly process, with few economic incentives for the pharmaceutical industry. Since 2014, only 18 new antibiotics have been approved, highlighting the slow progress despite rising resistance threats [8]. Antibiotic research and development require substantial investment in financial resources and time, which discourages large companies from investing in this field. The rapid evolution of resistance—particularly in *P. aeruginosa* and methicillin-resistant *Staphylococcus aureus* (MRSA)—can render new drugs ineffective within short timeframes, increasing urgency for alternatives [8,9].

The antibiotic crisis requires a coordinated global response, combining the search for new antimicrobials with robust resistance prevention and control policies. Developing new therapies must go hand-in-hand with antimicrobial stewardship programs to ensure prudent and responsible antibiotic use. Crucially, sustained funding and government support are needed to overcome the economic barriers in drug development. Integrating advanced technologies like artificial intelligence and machine learning with effective resistance management strategies could provide a sustainable solution to the AMR crisis, helping preserve effective bacterial treatments for future generations [7,11].

## 3. AMPs from Anurans as Therapeutic Alternatives for the Antibiotic Crisis

AMPs are defense molecules found in a wide variety of organisms, from bacteria to humans. These peptides play a crucial role in innate immunity, offering a first line of defense against microbial infections. They are typically short peptides, from 10 to 50 amino acids in length, and possess an amphipathic structure—having both hydrophilic and hydrophobic regions—that enables interaction with cell membranes. Most AMPs act directly on the membranes of pathogens, forming pores or destabilizing the membrane, leading to cell death. Some AMPs also interfere with protein synthesis, nucleic acid functions, or other essential intracellular pathways. In addition, many exhibit immunomodulatory activity [12,13,14].

In the context of the current antibiotic crisis, AMPs have gained prominence as potential alternatives or complements to conventional antibiotics. Given the growing global threat of AMR, AMPs are increasingly viewed as promising therapeutic options. AMPs act rapidly and are less likely to induce resistance, as they primarily target cell membranes—structures less susceptible to resistance-conferring mutations [15,16,17,18,19].

AMPs exert their effects through multiple mechanisms, including direct interaction with bacterial cell membranes, disruption of membrane integrity, and translocation into the cell to interfere with vital processes such as protein and nucleic acid synthesis [20].

Beyond their direct antimicrobial effects, AMPs exhibit a wide range of therapeutic properties, including antibiofilm, immunomodulatory, anti-inflammatory, wound-healing, and anticancer activities. Their ability to disrupt bacterial membranes makes them effective against multidrug-resistant pathogens, while their immunomodulatory functions help regulate host immune responses, reducing excessive inflammation [19,21].

**Figure 1:**
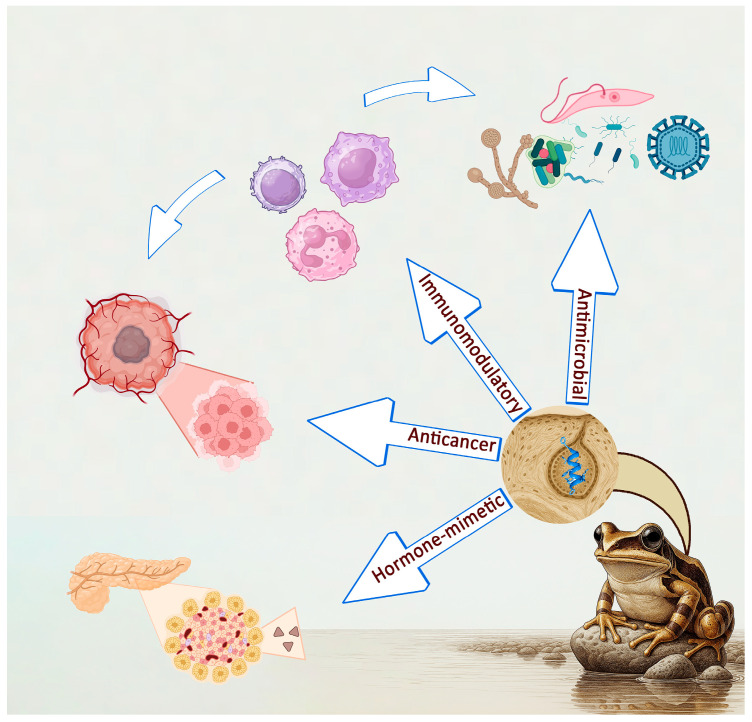
The main biological activities exhibited by anuran AMPs.

The skin secretions of amphibians, especially species from the order Anura, are rich sources of AMPs and have been the subject of intense research due to their therapeutic potential. Produced in the granular glands of the skin, these peptides serve as the first line of defense for anurans in pathogen-rich, humid environments. Many of these peptides have shown a broad range of therapeutic activities, including antimicrobial, anticancer, immunomodulatory, and metabolic regulatory effects, making them promising candidates for the development of new therapeutic agents [20,22,23,24,25,26,27,28] (Figure 1).

Several frog skin peptides enhance both innate and adaptive immunity by stimulating cytokine production in macrophages and lymphoid cells. Some, like frenatin 2D [29] and plasticin-L1 [30], lack direct antimicrobial effects but strongly activate macrophages. Others, including esculentin-2Cha [31], magainin-AM1 [32], and frenatins [33], exhibit both antimicrobial and immunostimulatory properties, making them promising pharmaceutical candidates.

AMPs promote tissue regeneration by enhancing cell migration and angiogenesis, making them valuable in chronic wound therapy [34]. Due to their small size and low production cost, amphibian-derived peptides are promising agents for treating chronic wounds. These peptides accelerate wound healing by promoting keratinocyte and fibroblast proliferation, immune cell recruitment, and immune response regulation [35]. Some, like MSI-1 [36] and brevinin-2PN [37], also exhibit antimicrobial activity, aiding infected wound healing. Their efficiency and defined mechanisms make them strong candidates for novel wound-healing therapies, offering potential for innovative treatments in regenerative medicine and antimicrobial applications.

Furthermore, some AMPs demonstrate selective cytotoxicity toward cancer cells [23,38]. For example, temporin-1CEa, an anticancer peptide from *Rana chensinensis*, binds negatively charged cancer cell membranes, inducing necrosis. Researchers designed 12 derivative peptides via double-cysteine scanning, with most showing enhanced anticancer activity. Nu-7 exhibited the strongest activity, possessing greater α-helicity than temporin-1CEa. Linked to podophyllotoxin, Nu-7-1 demonstrated superior anticancer effects, lower toxicity to normal HUVECs, and reduced hemolytic activity at therapeutic concentrations. Nu-7-1 damages cancer cell membranes and induces apoptosis. These modifications significantly enhanced anticancer activity and provide valuable insights for designing safer, more potent peptide-based therapies [39].

These multifunctional properties highlight their potential as versatile therapeutic agents in combating infections, chronic diseases, and even cancer.

Bioprospecting anuran skin secretions has yielded numerous peptides with promising therapeutic potential. Frogs, particularly those from the Leptodactylidae and Hylidae families, produce a wide range of peptides that serve as innate immune defense molecules, offering new templates for drug development [28,40]. These discoveries highlight the importance of biodiversity conservation, as many amphibian species are threatened by habitat loss and climate change [41,42]. Bioprospecting AMPs from anuran skin secretions involves a series of technical procedures to isolate, characterize, and evaluate their bioactivity. The process typically begins with non-invasive skin secretion collection, often induced by mild electrical stimulation or norepinephrine injection—both ethically approved methods that trigger peptide release. The crude secretion is then subjected to fractionation using high-performance liquid chromatography (HPLC), allowing for the separation of individual peptides based on their physicochemical properties such as hydrophobicity and charge [43,44]. Once isolated, peptide sequences are determined via mass spectrometry (MS) and Edman degradation, enabling structural identification [45]. De novo sequencing approaches, combined with bioinformatics tools, facilitate the classification of novel AMPs and their relationship with known antimicrobial sequences. Functional characterization includes antimicrobial assays against clinically significant pathogens, evaluating the minimum inhibitory concentration (MIC) and bactericidal effects, including activity against biofilms. Additionally, hemolysis and cytotoxicity assays assess selectivity toward microbial cells over mammalian cells, ensuring therapeutic potential [25,46]. Advancements in peptide synthesis and engineering further enhance AMP stability, biological activity, and bioavailability, contributing to drug development efforts [47,48,49,50,51]. Integrating these methodologies ensures a systematic and efficient approach to discovering novel therapeutic peptides from amphibians.

Constructing cDNA libraries from anuran skin secretions enables the cloning and characterization of AMP-encoding genes, facilitating the discovery of peptides with antimicrobial and antiviral properties. This approach has been successfully used to isolate and identify promising AMPs with therapeutic properties. For example, studies involving peptidomics and genomics analyses of *Odorrana grahami* identified 372 cDNA sequences encoding 107 novel AMPs across 30 families, including 24 new groups. This unprecedented diversity, driven by genetic mutations and recombination, challenges AMP redundancy in host defense. The peptides displayed diverse structures and antimicrobial mechanisms, including membrane disruption and DNA condensation. Forty AMPs were synthesized, revealing potential as antibiotic templates and emphasizing amphibian innate immunity [52]. Another relevant study is that by Ma et al. (2023) [53], which identified a dermaseptin-SS1 (SS1), a novel AMP, from *Phyllomedusa tarsius* skin secretions using a cDNA library and ‘shotgun’ cloning. Chemically synthesized, SS1 displayed broad activity against Gram-negative bacteria with low hemolytic potential. Its analog, 14V5K, demonstrated faster killing kinetics and lower salt sensitivity. Both peptides targeted bacterial membranes and displayed significant antiproliferative activity against lung cancer cells, highlighting their therapeutic potential.

**Figure 2:**
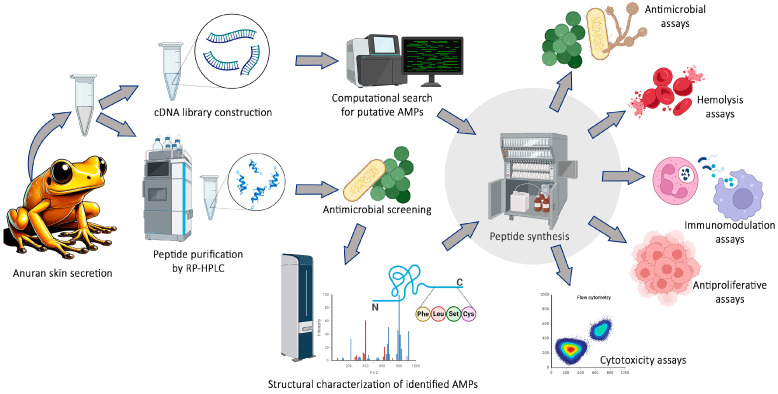
Workflow for the identification and characterization of novel AMPs.

The application of these techniques (Figure 2) can accelerate the identification of new AMPs, providing new treatment options and contributing to biodiversity conservation while offering innovative therapeutic alternatives.

**Figure 3:**
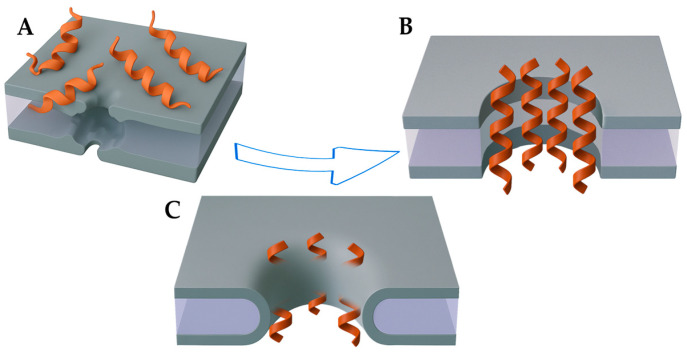
Membrane mode of action of AMPs. (**A**) Carpet-like model. (**B**) Barrel-stave model. (**C**) Toroidal pore model.

AMPs are generally classified by their secondary structures: β-sheets, α-helices, and linear or extended peptides. Most AMPs are cationic and amphipathic, properties that facilitate their interaction with microbial membranes [54,55]. The structural properties of AMPs largely dictate their membrane-targeting mechanisms of action. These mechanisms of action include the barrel-stave, toroidal pore, and carpet-like models described below (Figure 3).

### 3.1. Barrel-Stave Model

In the barrel-stave model, AMPs disrupt bacterial membranes by forming transmembrane pores. This model involves three key steps: (1) binding of monomers to the membrane, (2) insertion into the membrane to form pores, and (3) recruitment of additional monomers to expand the pore [56,57]. In the barrel-stave model, peptides insert perpendicularly into the lipid bilayer, and the subsequent recruitment of additional peptides leads to the formation of a transmembrane pore lined by these peptides. In this pore structure, the peptides are arranged with their hydrophobic sides facing the membrane’s lipid core and their hydrophilic regions oriented toward the pore’s interior. This configuration causes cellular contents to leak, ultimately resulting in cell death [58].

### 3.2. Toroidal Pore Model

The toroidal pore model shares structural similarities with the barrel-stave model but differs in its lipid–peptide interactions. In this model, monomers are also recruited, causing the pore to enlarge. Unlike barrel-stave pores, toroidal pores involve direct lipid–peptide cooperation: as AMPs insert into the membrane, they induce lipid curvature, continuously bending the monolayers inward. This results in a water-filled pore lined by both peptides and lipid headgroups, such as phosphatidylcholine. The peptides embed in the membrane, with hydrophobic regions interacting with lipid tails and hydrophilic regions stabilizing the pore interior. This process thins the membrane, reducing the energy required for pore formation and resulting in a configuration that allows for more fluid peptide-membrane interaction, increasing the efficiency of pore formation and subsequent cell lysis [57,58,59].

### 3.3. Carpet-like Model

In the carpet model, peptides interact electrostatically with the cell membrane: the positively charged regions bind to the negatively charged areas of the phospholipid layer. The peptides align parallel to the membrane surface, coating it in a carpet-like fashion. In this arrangement, the hydrophobic regions of the peptides contact the lipid core, while the polar faces interact with the charged groups of phospholipids, inducing curvature in the lipid bilayer. In the carpet model, the accumulation of AMPs on the membrane surface generates tension within the lipid bilayer. This tension destabilizes the membrane structure, ultimately leading to disruption of lipid packing, loss of membrane integrity, and formation of mixed peptide–lipid micelles. The process resembles a detergent-like mechanism, where high peptide density overwhelms the membrane, causing micellization and cell lysis [59].

### 3.4. Shai-Huang-Matsuzaki Model

The Shai–Matsuzaki–Huang model unifies key aspects of previous AMP mechanisms, proposing that AMPs act through dynamic, concentration-dependent interactions influenced by the properties of the membrane environment. According to this model, AMPs first accumulate on the membrane surface, where their cationic regions electrostatically bind to anionic phospholipids, while their hydrophobic domains integrate into the lipid bilayer. Initially, peptides align parallel to the membrane, but, upon reaching a critical concentration, they undergo a conformational shift, inserting perpendicularly into the bilayer. This structural rearrangement disrupts membrane integrity, forming transient toroidal pores—short-lived openings lined by peptides and lipid headgroups [60,61]. The model emphasizes the role of peptide-to-lipid ratio: at high concentrations, AMPs exhibit detergent-like behavior, leading to complete membrane dissolution, while, at lower concentrations, they induce transient perturbations, facilitating peptide translocation without immediate lysis. This translocation enables AMPs to access intracellular targets, including DNA, expanding their antimicrobial range beyond membrane disruption. The transient nature of these pores enhances peptide penetration, as frequent pore formation increases AMP translocation across the membrane. This process is influenced by peptide properties (e.g., size, charge, amphipathicity) and membrane composition. By integrating electrostatic interactions, amphiphilic insertion, and dynamic pore formation, the Shai–Matsuzaki–Huang model explains how AMPs selectively disrupt microbial membranes while enhancing selectivity toward microbial membranes, minimizing host cell toxicity [13,60,61].

Among the most studied AMP families from anurans, dermaseptins, brevinins, and temporins stand out. Dermaseptins, for example, were initially isolated from frogs of the genus *Phyllomedusa* and are known for their potent activity against Gram-positive and Gram-negative bacteria, as well as fungi and protozoa [40,62]. Brevinins, found in frogs of the genus *Rana*, exhibit high antimicrobial activity against Gram-positive and Gram-negative bacteria, as well as fungal pathogens [63]. Temporins are small AMPs (typically ranging from 10 to 13 amino acids) from frog skin, particularly from *Rana* species. They primarily targeted Gram-positive bacteria, including multi-resistant strains, by disrupting microbial membranes. Their immunomodulatory properties further enhance their value as potential therapeutic agents [38]. The diversity and efficacy of these peptides make them a promising source for the discovery of new antimicrobial drugs.

In summary, AMPs represent a diverse and powerful class of defensive molecules that play a crucial role in combating AMR. Their ability to act on different pathogens, combined with their low propensity to induce resistance, makes them ideal candidates for next-generation therapeutics. In the specific case of anurans, the AMPs found in their skin stand out for their broad activity and versatility, offering an abundant and underexplored source of new therapeutic agents at a critical time of global antibiotic crisis.

## 4. The Therapeutic Potential of AMPs from Anurans of the Genus *Boana*

Anurans of the genus *Boana* (formerly classified as *Hypsiboas*) were reclassified following a thorough taxonomic analysis by Dubois (2017) [64], which validated *Boana* as the correct scientific name for this group. The reclassification underscores an improved understanding of the diversity and phylogenetic relationships within the family Hylidae. Adopting the name *Boana* not only rectifies prior nomenclature but also emphasizes the critical role of ongoing taxonomic revisions in aligning species classification with their evolutionary history and biological diversity [64]. Accordingly, this review will also include peptides initially identified in *Hypsiboas* species that have since been reclassified as *Boana*.

*Boana* frogs are found across tropical and subtropical Americas, primarily inhabiting humid forests near water bodies. They show diverse morphology and ecology, with adhesive toe discs, long limbs, and color patterns often related to adaptations to their respective habitats [65,66]. *Boana* species display complex vocal behaviors during breeding to attract mates and defend territories [67].

Research on AMPs extracted from anuran skin has revealed a wealth of compounds with promising activity against bacterial, viral, and fungal pathogens. Among these, the genus *Boana* has emerged as a particularly rich source of AMPs.

The following section presents examples of peptides with therapeutic potential isolated from species of the *Boana* genus. The peptides are presented in chronological order, except when publications are directly related to the peptide under discussion.

Hylaseptin P1 (HSP1), a 14-residue amphipathic α-helical peptide from *Boana punctata* (previously *Hypsiboas punctatus*), showed comparable activity to commercial antibiotics: MICs of 6.1 µM for *S. aureus*, 24.4 µM for *Escherichia coli*, and 48.8 µM for *P. aeruginosa*. It also inhibited *Candida* spp. more effectively than fluconazole. At 195.2 µM (32× its MIC against *S. aureus*), it caused ≤5% hemolysis and showed minimal cytotoxicity on white cells or platelets in cytometric assays [68].

Junior et al. (2017) [69] developed a strategy to glycosylate HSP1-NH_2_ using solid-phase peptide synthesis and copper-catalyzed azide-alkyne cycloaddition, producing two glycotriazole-peptides and a triazole derivative. Biophysical assays confirmed stronger membrane disruption and lytic activity for glycotriazole-peptides. Biological tests showed enhanced antifungal activity and strong ergosterol biosynthesis inhibition in *Candida albicans*, attributed to membrane disruption (saccharide ring) and ergosterol inhibition (triazole and monosaccharide presence). These findings highlight glycotriazole-peptides as promising antifungal agents.

**Figure 4:**
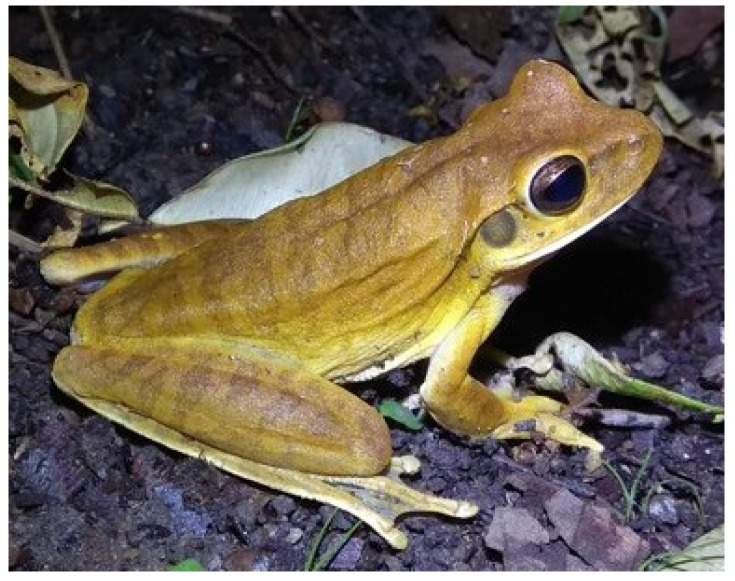
Adult specimen of *Boana raniceps*. Photo by Carlos José Correia de Santana.

A new family of AMPs, Raniseptins, was identified in the skin secretion of *Boana raniceps* (previously *Hypsiboas raniceps*) [70] (Figure 4). Nine cDNAs were cloned, sequenced, and peptides characterized. Raniseptins resemble dermaseptins in structure and are post-translationally cleaved into inactive fragments, likely to reduce toxicity or serve defensive functions. Raniseptin-1 (Rsp-1), the most abundant raniseptin in *B. raniceps* skin secretions, showed antimicrobial activity against *E. coli* (MIC = 5 µM), *P. aeruginosa* (MIC = 10 µM), and *S. aureus* (MIC = 20 µM), comparable to dermaseptins. Its fragments had weak or no activity, except against *Xanthomonas axonopodis* pv. *citri*. (MIC = 5 µM for Rsp-1(1–14)). The Rsp-1 fragments showed negligible hemolytic activity, while full-length Rsp-1 caused 20% hemolysis at 80 µM. MALDI imaging revealed intact peptide storage in glandular domains, with post-secretory cleavage generating inactive fragments, suggesting a role in predator defense rather than antimicrobial action.

Popov et al. (2019) [71] isolated three peptides (AC12, DK16, and RC11) from *B. raniceps* skin secretion. RC11 showed antimicrobial activity against *E. coli*, while AC12 and DK16 had no effect. AC12 was non-cytotoxic to RAW 264.7 cells, whereas DK16 and RC11 reduced cell viability. Hemolysis was minimal for AC12 and RC11, but DK16 demonstrated high hemolytic activity. AC12 and DK16 significantly reduced TNF-α and IL-12, with DK16 showing stronger effects. Both peptides also lowered IL-10 levels. Additionally, AC12 and DK16 inhibited NO production in a dose-dependent manner. In the in vivo carrageenan-induced paw edema assay, AC12 effectively reduced inflammation and edema, similar to dexamethasone. In the carrageenan-induced peritonitis assay, AC12 and RC11 reduced TNF-α, while AC12 also increased IL-10. Overall, AC12 was the most effective in reducing inflammation, highlighting its therapeutic potential.

Figainin 1, a novel peptide from *B. raniceps*, showed strong antibacterial activity against *E. coli*, *K. pneumoniae*, *S. aureus*, *S. epidermidis*, *Enterococcus faecalis*, and *E. casseliflavus* (with MICs ranging from 2 μM to 16 μM). Although Figainin 1 was ineffective against *P. aeruginosa* and *Candida*, it exhibited antiparasitic activity (*Trypanosoma cruzi epimastigotes*, IC_50_ = 15.9 µM) and cytotoxicity against both fibroblasts and multiple cancer cell lines (B16F10, MCF-7, HeLa). Despite its toxicity to noncancerous cells, Figainin 1 holds potential for developing novel anticancer and anti-infective agents [46].

A study on the multifunctional peptide Figainin 2, also derived from the frog *B. raniceps*, revealed broad-spectrum biological activities, including antimicrobial, antiparasitic, antiviral, and immunomodulatory effects. The peptide demonstrated potent antibacterial activity against both Gram-negative and Gram-positive bacteria, notably including multidrug-resistant *K. pneumoniae*, with particularly strong efficacy against *S. epidermidis* and *E. casseliflavus* (though inactive against *Candida* spp.). Figainin 2 exhibited significant antiparasitic activity against *T. cruzi* epimastigotes (IC_50_ = 6.32 µM) and displayed moderate hemolysis (23% at 32 µM). It also showed promising anticancer effects, inhibiting murine melanoma (B16F10, IC_50_ = 12.8 µM) and human breast cancer cells (MCF-7, IC_50_ = 15.3 µM). Additionally, Figainin 2 exhibited dose-dependent antiviral activity against CHIKV, DENV4, and YFV (EC_50_ = 17–21.8 µM) without cytotoxicity to noninfected Huh7 cells at 25 µM. At 8 µM, it stimulated reactive oxygen species (ROS) production in human neutrophils, comparable to the bacterial peptide fMLP [43]. These findings position Figainin 2 as a candidate for development in anti-infective, anticancer, and immunomodulatory therapies—pending optimization to reduce hemolytic effects.

Raniseptins-3 and -6 are cationic peptides from *B. raniceps* skin secretions that adopt α-helical conformations in membrane-mimetic environments, consistent with membrane-disruptive mechanisms. They showed strong antibacterial activity, particularly against *E. coli*, *K. pneumoniae*, and a carbapenem-resistant *K. pneumoniae* strain, with MICs as low as 1–4 µM. Raniseptin-3 was notably more effective against *S. aureus* than Raniseptin-6. Despite high concentrations, neither peptide effectively inhibited *C. albicans*. Hemolytic assays demonstrated low toxicity toward human erythrocytes at therapeutic levels (<5% hemolysis). However, both peptides showed cytotoxic effects against murine fibroblasts (NIH3T3) and melanoma cells (B16F10), with low IC_50_ values. In antiviral assays against MHV-3, a murine hepatitis virus that shares the same genus (*Betacoronavirus*) with the SARS-CoV-2 virus, neither peptide improved cell viability or reduced viral cytotoxicity. These findings underscore their antimicrobial and anticancer potential [45].

The skin secretion of *Boana albopuctata* (previously *Hypsiboas albopunctatus*) led to the isolation of Hylin a1, a cytolytic 18-residue amidated peptide. Hylin a1 displayed broad-spectrum antimicrobial activity, particularly against Gram-positive bacteria (*S. aureus*, *E. faecalis*, *B. subtilis*, MICs: 8–16 μM) and was also active against *Candida* species and *Cryptococcus neoformans*. It also exhibited high hemolytic activity (HC_50_ = 18 μM) [72].

Hylin a1’s antitumor effects were evaluated, and its targeting was enhanced by RGD conjugation, forming RGD-hylin a1, which was loaded onto mesoporous silica (HMS-COOH). The RGD-hylin a1-HMS system induced cancer cell apoptosis at pH 5.5, remained harmless at pH 7, and reduced hemolysis by 50–100%. In tumor-bearing mice, it inhibited tumor growth by 50–60%, highlighting its potential as a targeted antitumor therapy [73].

The antibacterial activity of Hylin-a1 against *S. aureus* multi-resistant strains was assessed. Hylin-a1 exhibited bacteriostatic effects, inhibiting 90% of bacterial growth at 6.25 μM, with enhanced potency (~3 μM) against β-lactam- and methicillin-resistant strains. It modulated inflammatory cytokines (IL-1β, IL-6, IL-8) and altered *S. aureus* cell morphology, suggesting therapeutic potential for diverse *S. aureus* infections, including resistant strains [74].

Hylin-a1 was found to inhibit the entry of several viruses, including canine distemper virus (CDV, *Paramyxoviridae*), bovine viral diahrrea virus (BVDV, *Flaviviridae*), Schmallenberg virus (SBV, *Bunyaviridae*), and animal herpesviruses, namely bovine herpesvirus type 1 (BoHV-1) and caprine herpesvirus type 1 (CpHV-1). It likely acts through physical interaction with the hydrophobic viral surfaces. Further in vitro and in vivo studies are required to assess its clinical potential for human application [75].

In another study, Hylin-a1 demonstrates broad-spectrum antiviral activity against enveloped respiratory viruses (SARS-CoV-2, HCoV-229E, MeV, HPIV-3, RSV, influenza) by disrupting viral envelopes. Its ability to irreparably damage these pathogens suggests potential as a pan-inhibitor, offering a promising tool to combat current and future pandemics caused by enveloped viruses [76].

Recently, Chianese et al. (2024) [77] described that Hylin-a1 inhibits herpes simplex virus type 1 (HSV-1) and type 2 (HSV-2) by disrupting viral envelopes at early infection stages, including acyclovir-resistant strains. With low hemolysis, anti-inflammatory effects, serum stability, and synergistic action with acyclovir, Hylin-a1 emerges as a promising clinical antiherpetic agent.

Another study conducted by Siano et al. (2014) [78] identified and characterized several AMPs in the skin of *Boana pulchella* (previously *Hypsiboas pulchellus*) using LC-MS-MS. Twenty-three novel sequences were identified, with three peptides selected for synthesis: P1-Hp-1971, P2-Hp-1935, and P3-Hp-1891. These peptides inhibited the growth of *E. coli* and *S. aureus*, with P1-Hp-1971 and P3-Hp-1891 showing the highest activity. P1-Hp-1971 exhibited the best therapeutic indices, particularly against *S. aureus*.

Nacif-Marçal et al. (2015) [79] isolated and characterized the peptide Hs-1 from the skin of *Boana semilineata* (previously *Hypsiboas semilineatus*). Hs-1 showed selective antimicrobial activity against Gram-positive bacteria, with a MIC range from 11 to 46 μM, but it did not show any effect against Gram-negative bacteria. Transmission electron microscopy analysis revealed that Hs-1 affects bacterial cells in a distinct manner, suggesting a specific mechanism of action.

In addition to antibacterial applications, the antiviral activity of peptide Hs-1 has also been a focus of research. Monteiro et al. (2018) [80] found that it effectively inhibits infection by dengue virus serotypes 2 and 3, demonstrating promising antiviral potential. The Hs-1 peptide inhibited dengue-2 (DENV-2) and dengue-3 (DENV-3) infection in adsorption assays (90–100%, dose-dependent). Internalization assays showed 95–100% inhibition for DENV-2 and 80–90% for DENV-3. qPCR confirmed fewer viral genome copies, especially for DENV-2. Hs-1 likely prevents viral attachment by neutralizing heparan sulfate. In vivo, Hs-1 provided 100% protection against DENV-2 and 40% against DENV-3, supporting viral envelope disruption as its mechanism. Further studies on other serotypes are needed for broader efficacy [80].

A novel peptide family named Pugnins was discovered by Liscano et al. (2021) [81] in the skin transcriptome of *Boana pugnax*, displaying dual antibacterial and anticancer activities. Molecular docking and dynamics simulations revealed strong interactions between Pugnins and bacterial membranes, as well as anticancer targets, suggesting their potential as therapeutic agents. Experimental studies verified their effectiveness against both Gram-positive and Gram-negative bacterial strains with MIC values ranging from 2 µM to 16 µM. The peptides also showed promising cytotoxicity against cancer cell lines, suggesting anticancer potential. While Pugnins displayed moderate hemolytic effects, the findings emphasize the value of amphibian-derived peptides in therapeutics and demonstrate how computational methods can accelerate the discovery of bioactive drug candidates.

Another example is the work by Nunes et al. (2021) [82], who investigated the peptide Hylaseptin-4, an AMP isolated from *B. punctata*, which displays a well-defined α-helical conformation in biomimetic environments, forming aggregates at elevated concentrations. The peptide demonstrates pH-dependent antibacterial activity, with MIC values of 22 μM against both *E. coli* and *S. aureus*, and 44 μM against *P. aeruginosa* at acidic to neutral pH (5.0–6.7). Notably, its antimicrobial efficacy diminishes at pH 8.0, corresponding to reduced helical stability. Molecular dynamics simulations indicate that self-aggregation may contribute to both structural integrity and enhanced antimicrobial activity under physiological conditions.

In *Boana picturata*, new families of AMPs, such as picturins and pictuseptins, were identified. Pictuseptins, in particular, demonstrated broad antimicrobial and antifungal activity, with low hemolytic activity, suggesting good selectivity [83].

Another study focused on *Boana boans* revealed the presence of host defense peptides with therapeutic potential, including figainin 2BN, which exhibited strong antimicrobial and cytotoxic activity, albeit without cancer cell selectivity—limiting therapeutic application [84].

The natural peptide BcI-1003 from *Boana cordobae* shows promise as a multi-target therapeutic agent for Alzheimer’s disease (AD). It inhibits BChE (butyrylcholinesterase, IC_50_ = 669 μM) and MAO-B (monoamine oxidase B, IC_50_ = 570 μM) and shows potent antioxidant activity (EC_50_ = 7.24 μM). BcI-1003 also acts against drug-resistant bacteria, particularly *E. coli* MDR-1 (MIC = 8 μM), and is non-toxic to human erythrocytes. These properties support its potential for treating AD and microbial infections [85].

Studies with *Boana platanera* identified multifunctional host defense peptides that exhibited rapid bactericidal and cytotoxic activities against human tumor cells, as well as stimulated insulin release, suggesting their potential as new anti-infective and antidiabetic agents. Five host-defense peptides (figainin 2PL, hylin PL, raniseptin PL, plasticin PL, and peptide YL) were isolated from the skin secretions of the frog *Boana platanera*. Raniseptin PL and figainin 2PL exhibited strong bactericidal activity against ESKAPE pathogens and *Clostridioides difficile*, as well as potent cytotoxic effects on human tumor cell lines (A549, MDA-MB-231, and HT29) with low hemolytic activity against mouse erythrocytes. The peptides hylin PL, raniseptin PL, and peptide YL enhanced insulin release from β-cells, with peptide YL showing the greatest effect, suggesting its potential for developing new anti-diabetic drugs [86].

Table 1 presents the peptides isolated from the skin secretion of anurans of the genus *Boana* described in this review article, along with their primary structures, charge and main biological activities.

## 5. Concluding Remarks

Exploring anuran biodiversity for novel AMPs is essential in the face of rising global AMR. Secreted through the skin, these peptides display broad antimicrobial and antiviral activity, positioning them as strong candidates for next-generation therapeutics. Studies highlight their effectiveness against drug-resistant bacteria and emerging viral threats, including SARS-CoV-2 [27,28].

The chemical diversity of anuran-derived AMPs presents a valuable pool of molecules with varied mechanisms of action, offering potential breakthroughs in treating infections that current antibiotics struggle to manage. Beyond antimicrobial action, many AMPs modulate immune responses and promote tissue repair, making them attractive candidates for treating inflammatory and infectious conditions [35,87]. These multifunctional properties support their use in therapies for cancer, metabolic disorders, and chronic inflammation—beyond infection control.

Realizing their full therapeutic potential depends on systematic bioprospecting, peptide characterization, and mechanistic studies. Investment in this research not only advances pharmacological innovation but also promotes biodiversity conservation by highlighting the ecological importance of these species, since preserving amphibian biodiversity is not only vital for ecosystem health, but also fuels pharmacological innovation through AMP discovery.

Recent studies on *Boana* species underscore their value as sources of bioactive peptides with diverse therapeutic potential. *Boana*-derived AMPs represent a powerful and versatile class of bioactive molecules with demonstrated efficacy against pathogens, inflammation, and tumor cells and represent a promising foundation for developing alternative therapies. Integrating structural and functional studies is essential to harness their full potential in addressing microbial resistance and unmet medical needs.

## Figures and Tables

**Table 1:** AMPs isolated from the skin secretion of *Boana* species.

Peptide	Sequence	Species	Biological Activity	Charge ^a^	Reference
Hylaseptin P1	GILDAIKAIAKAAG ^b^	*Boana punctata*	Antibacterial Antifungal	+1	[68]
Raniseptin-1	AWLDKLKSLGKVVGKVALGVAQNYLNPQQ	*Boana raniceps*	Antibacterial	+3	[70]
AC12	ACFLTRLGTYVC-NH_2_	*Boana raniceps*	Immunomodulatory	+2	[71]
DK16	DKERPICSNTFRGRKC-NH_2_	*Boana raniceps*	Immunomodulatory	+4	[71]
RC11	RCFRRRGKLTC-NH_2_	*Boana raniceps*	Antibacterial Immunomodulatory	+6	[71]
Figainin 1	FIGTLIPLALGALTKLFK-NH_2_	*Boana raniceps*	Antibacterial Anti-*T. cruzi* Anticancer	+3	[46]
Figainin 2	FLGAILKIGHALAKTVLPMVTNAFKPKQ	*Boana raniceps*	Antibacterial Anti-*T. cruzi* Anticancer Antiviral Immunomodulatory	+4	[43]
Raniseptin-3	AWLDKLKSIGKVVGKVAIGVAKNLLNPQ	*Boana raniceps*	Antibacterial Anticancer	+4	[45]
Raniseptin-6	ALLDKLKSLGKVVGKVALGVVQNYLNPRQ	*Boana raniceps*	Antibacterial Anticancer	+4	[45]
Hylin a1	IFGAILPLALGALKNLIK-NH_2_	*Boana albopunctata*	Antibacterial Antifungal Antiviral Immunomodulatory	+3	[72,74,75,76,77]
P1-Hp-1971	TKPTLLGLPLGAGPAAGPGKR-NH_2_	*Boana pulchella*	Antibacterial	+4	[78]
P2-Hp-1935	KLSPSLGPVSKGKLLAGQR-NH_2_	*Boana pulchella*	Antibacterial	+5	[78]
P3-Hp-1891	RLGTALPALLKTLLAGLNG-NH_2_	*Boana pulchella*	Antibacterial	+3	[78]
Hs-1	FLPLILPSIVTALSSFLKQG-NH_2_	*Boana semilineata*	Antibacterial Antiviral	+2	[79,80]
Pugnin A	RLMRIFRILKLAR	*Boana pugnax*	Antibacterial Anticancer	+5	[81]
Pugnin B	RMMRIFWVIKLAR	*Boana pugnax*	Antibacterial Anticancer	+4	[81]
Hylaseptin-4	GIGDILKNLAKAAGKAALHAVGESL-NH_2_	*Boana punctata*	Antibacterial	+2	[82]
Picturin-1	GVFKDALKQLGAALLDKAANALKPK	*Boana picturata*	Antibacterial Antifungal	+3	[83]
Picturin-2	GVFKDALKQFGAALLDKAANALKPK	*Boana picturata*	Antibacterial Antifungal	+3	[83]
Picturin-3	GVFKDALKQFGAALLDQAANALKPK	*Boana picturata*	Antibacterial Antifungal	+2	[83]
Pictuseptin-1	GFLDTLKNIGKTVGRIALNVLT-NH_2_	*Boana picturata*	Antibacterial Antifungal	+3	[83]
Pictuseptin-2	GFLDTLKNIGKTVGGIALNVLT-NH_2_	*Boana picturata*	Antibacterial Antifungal	+2	[83]
Pictuseptin-3	GFLDTLKNIGKTVGKVALDVAKNVLT-NH_2_	*Boana picturata*	Antibacterial Antifungal	+3	[83]
Figainin 2BN	FLGVALKLGKVLGKALLPLASSLLHSQ	*Boana boans*	Antibacterial Anticancer	+3	[84]
Picturin 1BN	GIFKDTLKKVVAAVLTTVADNIHPK	*Boana boans*	Antibacterial Anticancer	+2	[84]
Picturin 2BN	GLMDMLKKVGKVALTVAKSALLP	*Boana boans*	Antibacterial Anticancer	+3	[84]
BcI-1003	GSKKTKCPR-NH_2_	*Boana cordobae*	Antibacterial Antioxidant BChE inhibition MAO-B Inhibition	+5	[85]
Raniseptin PL	GVFDTVKKIGKAVGKFALGVAKNYLNS-NH_2_	*Boana platanera*	Antibacterial Anticancer Antidiabetic	+5	[86]
Figainin 2PL	FLGTVLKLGKAIAKTVVPMLTNAMQPKQ-NH_2_	*Boana platanera*	Antibacterial Anticancer	+5	[86]
Hylin PL	FLGLIPALAGAIGNLIK-NH_2_	*Boana platanera*	Antidiabetic	+2	[86]
Peptide YL	YVPGVIESLL-NH_2_	*Boana platanera*	Antidiabetic	0	[86]

^a^ Net charge at pH 7.0. Calculated with the peptide calculator tool at https://www.bachem.com/knowledge-center/peptide-calculator/ (accessed on 4 June 2025). ^b^ Unless mentioned, peptides exhibit free C-terminus (-COOH).

## Data Availability

No new data were created or analyzed in this study.

## Abbreviations

The following abbreviations are used in this manuscript:

AI	Artificial intelligence
AD	Alzheimer’s disease
AMPs	Antimicrobial peptides
AMR	Antimicrobial resistance
cDNA	Complementary deoxyribonucleic acid
HC_50_	Hemolytic concentration 50
HPLC	High-performance liquid chromatography
MIC	Minimum inhibitory concentration
MS	Mass spectrometry
WHO	World Health Organization
